# Catecholaminergic Polymorphic Ventricular Tachycardia Presented As Generalized Tonic-Clonic Seizure: A Case Report

**DOI:** 10.7759/cureus.27806

**Published:** 2022-08-09

**Authors:** Shraddha Acharya, Pratichhya Devkota, Ramesh Shrestha, Ashik K Bajracharya, Stephen Jesmajian

**Affiliations:** 1 Internal Medicine, Montefiore New Rochelle Hospital, New York, USA; 2 Cardiology, Heart & Vascular Institute, Detroit, USA

**Keywords:** ryanodine receptor, long qt, sudden cardiac arrest, seizure, polymorphic ventricular tachycardia, catecholaminergic

## Abstract

Catecholaminergic polymorphic ventricular tachycardia (CPVT) is an inherited, highly malignant cardiac channelopathy that causes autopsy-negative sudden deaths and sudden infant deaths. The symptoms of CPVT range from asymptomatic to syncopal. We present a patient who has had sporadic seizures for the last four years and was diagnosed with focal seizures. Genetic testing revealed heterozygosity for a variant of uncertain significance in the cardiac ryanodine receptor (RYR2). Pathogenic variants are known to be associated with CPVT. A subcutaneous implantable cardioverter-defibrillator (ICD) was placed and is being closely followed in the cardiology clinic.

## Introduction

Catecholaminergic polymorphic ventricular tachycardia (CPVT) is a highly malignant, inherited cardiac channelopathy [1]. It is associated with potentially life-threatening catecholamine-mediated ventricular arrhythmias (VAs) triggered by stress or exertion. It accounts for 12% of autopsy-negative sudden deaths and 1.5% of sudden infant deaths, although the true prevalence is unknown [2,3]. CPVT has a variable presentation, ranging from asymptomatic to syncope, dizziness, palpitations during strenuous activities, or sudden death [4]. In this report, we present the case of a young male with seizures who presented with VAs and was subsequently found to have CPVT.

## Case presentation

A 22-year-old male presented to the emergency department (ED) after having a seizure at work six hours earlier. It was described as tonic-clonic, lasting about one to two minutes, and the patient did not hit his head or sustain any other injuries. He was lethargic afterward. He had nausea with several episodes of vomiting upon waking up. He was then brought to the ED by Emergency Medical Service. During the exam, he was somnolent with easy arousal.

The only prodromal symptom reported by the patient was dizziness. During his episodes, he has lost consciousness. He has no recollection of having palpitations, shortness of breath, chest pain, or incontinence. There was no reported history of seizures, cardiac disease, or sudden death in the family. For the last four years, the patient has had three to four sporadic seizure episodes and was subsequently diagnosed with focal seizures. He was started on low-dose levetiracetam which was titrated up after another episode of seizure one year ago. The previous brain MRI was normal, and a 72-hour ambulatory electroencephalogram (EEG) did not reveal any seizure events.

**Figure 1:**
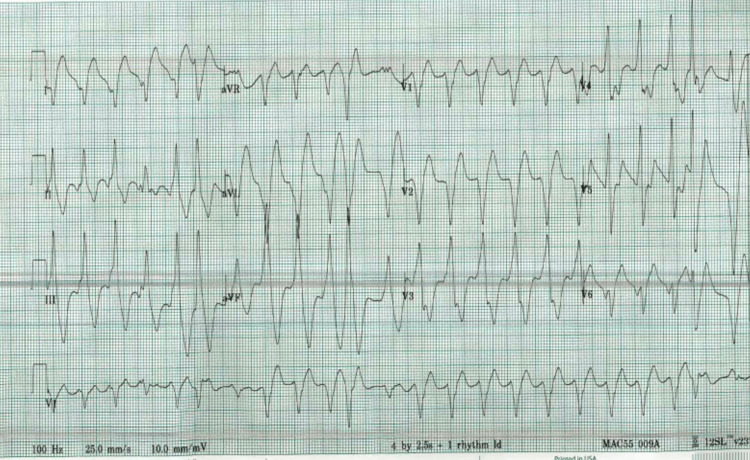
Wide complex ventricular tachycardia

His vital signs were stable when he arrived at the ED. Physical exams including an extensive neurological exam were normal. The laboratory parameters were unremarkable. Four hours later, he had another seizure that lasted one minute and was treated with 2 mg of lorazepam and 1,000 mg of levetiracetam. During the second seizure episode, an electrocardiogram (ECG) was obtained which revealed wide complex ventricular tachycardia (VT) (Figure 1). When the seizure episode ended, the PVT subsided and no cardiac defibrillation was required. After the return of spontaneous circulation, vitals were as follows: blood pressure 110/70 mmHg, respiration rate 12/min, pulse rate 88 beats per minute, temperature 98.7°F, and 95% oxygen saturation in the nasal cannula.

Cardiology was consulted, and cardiac structural studies (echocardiogram and cardiac MRI) and electrophysiology (EP) studies were recommended. An echocardiogram revealed no significant abnormalities. Cardiac MRI revealed normal biventricular systolic function, mild biventricular dilatation, and a small non-specific focal point of late gadolinium enhancement in the inferior right ventricle. EP studies revealed no inducible arrhythmias (supraventricular tachycardia or VT). However, intermittent self-terminating brief atrial flutter (AF) and a brief left-sided isorhythmic self-terminated polymorphic VT episode were noted during the exercise stress test.

During the acute VT episodes, propranolol was used; however, maintenance therapy was started with nadolol. Patient and family initially deferred subcutaneous implantable cardioverter-defibrillator (ICD) and proceeded with interim use of wearable cardiac defibrillator during the assessment of response to beta-blocker and additional discussions regarding ICD. The patient was not having any seizure activity; so after consulting with neurology, the antiepileptic drug, levetiracetam, was tapered off. Because the patient was concerned about having another seizure episode, a low dose of oxcarbazepine was continued. A few months later, a subcutaneous ICD was implanted, which is less invasive and the newest type.

Outpatient genetic testing revealed heterozygosity for a variant of unknown significance in the cardiac ryanodine receptor (RYR2), a gene where pathogenic variants have been linked to CPVT. The variant's in silico analysis indicated that it was most likely pathogenic. Without further episodes of seizure or abnormal cardiac activity, the patient is currently compliant with medications. Anti-epileptic medications were continued for the time being. He is being closely monitored in the cardiology clinic.

## Discussion

Polymorphic ventricular tachycardia (VT) is described as a ventricular rhythm (>100 beats per minute) with a continuously changing QRS complex morphology in any recorded electrocardiographic (ECG) lead [5]. CPVT, also known as familial CPVT, occurs in the absence of structural heart disease or known associated syndromes [6]. CPVT, long QT syndrome, and Brugada syndrome are three conditions that commonly lead to sudden cardiac death (SCD) [4]. It is distinguished from the other two by the absence of abnormal resting ECG (long QT or Brugada pattern, respectively) [7]. Bidirectional polymorphic tachycardia and ventricular arrhythmias that are reproduced by exercise or intense emotion are hallmarks of CPVT [8]. The autosomal dominant RyR2 gene for the cardiac ryanodine receptor or the autosomal recessive CASQ2 gene for calsequestrin 2 accounts for 70% of the genetic mutations, and both have high penetrance [9]. Both mutations appear to work by causing the sarcoplasmic reticulum to release calcium during diastole. The resulting intracellular calcium overload leads to delayed afterdepolarizations and triggered activity, which can induce ventricular tachycardia and fibrillation [7].

The prevalence in the general population has been estimated to be at one in 10,000, with a mortality rate of up to 50% in severe, untreated cases [10]. It commonly presents in children between three and 16 years of age, with exercise syncope or sudden cardiac arrest (SCA), which is precipitated by physical or emotional stress including swimming [11]. However, first presentations in later adulthood are not uncommon. As in our case, syncope can present with convulsions and can be misdiagnosed with epilepsy. RYR2 mutations are also linked with focal seizures, which can further complicate the diagnosis [12]. Family history of syncope is common, and 26.5% of patients have family members with SCD before age 14.2 ± 10.9 years [13].

Diagnosis can be challenging due to normal inter-episode ECG and no distinct changes in imaging. As in this case, the key to diagnosis is capturing abnormal ECG either during an episode or in stress testing [2]. When CPVT patients start exercising, ventricular ectopy develops, increasing in complexity as the heart rate rises, with QRS alternating by 180 degrees on a beat-by-beat basis [14]. This bi-directionality, while considered a hallmark of CPVT, can occur with other conditions such as digoxin toxicity [3,10]. Holter monitoring can be considered in patients who are unable to have adequate exercise stress but have lower sensitivity [3].

It is recommended that all clinically and genetically diagnosed CPVT patients be treated. Stopping an acute polymorphic VT episode, preventing cardiac arrest and sustained VT with an implantable cardioverter-defibrillator (ICD) and antiadrenergic medications, and minimizing VT recurrence are the basic principles of management. Beta-blockers are used in the acute and maintenance phases of CPVT. All patients are started on beta-blockers after initial stabilization [3,15]. For acute suppression of recurrent polymorphic VT, we use propranolol 40 mg oral doses or appropriate weight-based dosing in children, every six hours for the first 48 hours, with additional intravenous doses as needed for recurrent breakthrough ventricular arrhythmias. Long-term preventive therapy with nadolol 1 to 2 mg/kg is preferred (because of its long duration of action) [4]. Flecainide (medium daily dose of 150 mg) has been shown to significantly lower ventricular arrhythmias during exercise when added to baseline therapy with a beta-blocker [16]. We advise using verapamil as an additional treatment for CPVT patients who continue to experience ventricular arrhythmias despite taking beta-blockers and/or flecainide [17]. Since beta-blockers and ICDs are efficient treatments for CPVT and SCD risk is present in 50% of cases, early diagnosis and subsequent preventive measures are made possible by genetic screening for the RYR2 mutation [18].

## Conclusions

Catecholaminergic polymorphic ventricular tachycardia (CPVT) is a highly malignant, inherited cardiac channelopathy. CPVT is an arrhythmogenic condition that is frequently misdiagnosed. The presentation of CPVT varies, from asymptomatic to syncopal. A high index of suspicion for CPVT must be entertained in young patients who collapse unexpectedly in the setting of exercise or under intense emotional stress. A non-selective beta-blocker is the first-line treatment. Patients are advised to avoid physical or emotional triggers. Patients who present with sudden cardiac arrest due to adrenergic stimuli are frequently misdiagnosed with a seizure disorder or vasovagal syncope. CPVT should be considered in the differential diagnosis of patients who present with recurrent epileptic episodes caused by exertion.
